# The Performance of Visual Perceptual Tasks in Patients with Schizotypal Personality Disorder

**DOI:** 10.11621/pir2021.0204

**Published:** 2021-06-30

**Authors:** Anastasia A. Chepeliuk, Marina G. Vinogradova

**Affiliations:** a FSBI “Zakusov Institute of Pharmacology”, Moscow, Russia; b Sechenov First Moscow State Medical University (Sechenov University), Moscow, Russia; c Faculty of psychology, Lomonosov Moscow State University, Moscow, Russia

**Keywords:** personality disorders, schizotypal personality disorder, obsessive-compulsive personality disorder, cognitive functions, visual perceptual tasks, Wechsler Adult Intelligence Scale (WAIS-R)

## Abstract

**Background:**

The most significant features for clinical diagnosis of schizotypal personality disorder (SPD) are cognitive-perceptual and disorganized symptoms. Experimental study of visual perceptual processes is important to elucidate the psychological mechanisms of cognitive-perceptual impairment in SPD.

**Objective:**

To research the performance of visual perceptual tasks in SPD.

**Design:**

Series I and II presented the subjects with visual perceptual tasks with different types of instructions (vague, verbal, or visual perceptual cues). The Wechsler Adult Intelligence Scale (WAIS-R) was also administered. The participants were 39 SPD patients, 36 obsessive-compulsive personality disorder (OCPD) patients (F.21.8, F.60.5 in ICD-10, respectively), and 102 healthy controls.

**Results:**

SPD patients had a significantly lower number of correct answers in conditions of vague instruction and verbal cues in Series I of a visual-perceptual task in comparison with healthy subjects (*p* < 0.01). With visual perceptual cues in Series II, patients with SPD had the same number of correct answers as controls, whereas OCPD patients had the same number of correct answers as controls with verbal cues in Series I. SPD patients had significantly lower scores in most verbal and nonverbal WAIS-R subtests in comparison with controls. SPD patients differed from OCPD patients in that they had lower scores in the “Information” (*p* < 0.05) and “Comprehension” (*p* < 0.05) subtests.

**Conclusion:**

With visual-perceptual cues, SPD patients were able to achieve normative results in the performance of visual-perceptual tasks, whereas patients with OCPD demonstrated lower productivity. In SPD patients, the basic impairments were associated with difficulties in inhibition of peculiar responses, stability of a subjective manner of performance and inability to revise it, low orientation to the model, and slipping into subjective associations with the stimuli.

## Introduction

Psychiatric research on cognitive deficits in schizophrenia and in non-psychotic disorders within the schizophrenia spectrum is considered to be a promising approach for understanding mental dysfunction (Carruthers, Van Rheenen, Gurvich, Sumner, & Rossell, 2019; Rosell, Futterman, McMaster, & Siever, 2014). Schizotypal personality disorder (SPD) shares many biological features with schizophrenia and represents an intermediate schizophrenia-spectrum phenotype. This makes it possible to study mental disorders that do not reach the level of psychosis, to better understand the genetics, pathogenesis, and cognitive symptoms related to psychotic illnesses. At the same time, clinicians and researchers are uncertain about the boundaries on the continuum of personality pathology and psychotic pathology in SPD (Anderson & Sellbom, 2018; Smulevich, Romanov, Mukhorina, & Atadzhikova, 2017). There are different mechanisms and factors that could lead to a transition from constitutional traits to susceptibility to the development of psychotic disorders: neuroanatomical peculiarities (Dickey et al., 2005; Dickey, McCarley, & Shenton, 2002; Liu et al., 2016), genetic factors (Zouraraki, Karamaouna, Karagiannopoulou, & Giakoumaki, 2017), psycho-physiological mechanisms (Ahn, Lustenberger, Jarskog, & Fröhlich, 2020; Hazlett et al., 2015; Rabella et al., 2016), etc.

Cognitive dysfunction is an essential line of modern clinical and psychological research into schizophrenia-spectrum disorders as one of the levels of the disease (Rodriguez et al., 2019; Siddi, Petretto, & Preti, 2017). Moreover, cognitive impairment in SPD is considered a prognostic indicator for the development of schizophrenia. For instance, the severity of impairment of visual perceptual processes is associated with earlier onset of the disease, the development of delusions and hallucinations, bizarre behavior, depressive symptoms, and a low level of social functioning in childhood and adolescence. In addition, a number of disturbances of the visual-perceptual processes (altered perception of one’s face, body, the faces of other people, pseudo-movement, reversions, etc.) are stable and don’t depend on the duration of the disease or the type of pharmacotherapy (Keane, Cruz, Paterno, & Silverstein, 2018).

Clinicians suggest that the most significant clinical diagnostic criteria for SPD are cognitive-perceptual (magical ideation, unusual perceptual experiences) and bizarreness, disorganized criteria (for example, eccentric behavior and speech, inappropriate, restricted affect) (Zouraraki et al., 2017). A distinctive characteristic of cognitive-perceptual processes in SPD is their multidimensionality, not only dysfunctional manifestations, but also compensatory strategies. Psychological studies report that, on the one hand, cognitive and perceptual deficits in SPD facilitate an altered state of creativity and immersion into unusual perceptual experience; on the other, cognitive and perceptual peculiarities are an adequate way to compensate for the schizotypal maladaptation (Kallai et al., 2019). Recent studies also report the absence of manifestations of speech disorders in the aspect of originality (increasing the number of original answers), and semantic flexibility in subjects with high schizotypal traits (Rodríguez-Ferreiro & Aguilera, 2019).

Psychological studies on visual perceptual processes in SPD focus on several different aspects. The neuropsychological approach describes dysfunction of spatial working memory (Smith & Lenzenweger, 2013), executive functions, information processing (Giakoumaki, 2012), processing speed (McClure, Harvey, Bowie, Iacoviello, & Siever, 2013), and attention (Carrión et al., 2011; Dickey et al., 2005). The factors determining disturbances of visual perceptual processes, speech, and thinking in SPD include increased stress and uncertainty of visual perceptual stimuli (Silverstein & Palumbo, 1995). In these conditions, patients with SPD demonstrated diminished accuracy of visual spatial memory (Smith & Lenzenweger, 2013). Disorganization of speech is reported while patients view both pleasant and stressful photographs (Minor & Cohen, 2010). The role of negative affect (general psychological distress) in the occurrence of cognitive impairment in individuals with high schizotypy scores has also been discussed (Carrigan & Barkus, 2017).

Experimental studies of perception in patients with SPD have shown intact early visual perceptual processes and heightened mental imagery (Maróthi & Kéri, 2018), which probably contribute to perceptual aberration in SPD. One of the psychological mechanisms of unusual body experience (self-face perception abnormalities, loss of self-other boundaries) in schizophrenia spectrum disorders was demonstrated by using the Strange-Face-in-the Mirror illusion and Mirror-Gazing test (Bortolon et al., 2017; Caputo, 2010). The particular interest of modern studies of visual perceptual processes in schizophrenia spectrum disorders focuses on sensitivity to changes in target shape (for instance, target detection in symmetrical patterns and noise, configural superiority effect, global-local divided attention task, performance on closed-contour tasks). These studies indicate a specific difficulty with the perceptual organization of stimuli and heterogeneity of cognitive perceptual functioning in patients with schizophrenia and SPD (Panton, Badcock, & Badcock, 2016).

Social cognition is a significant facet of visual perceptual processing. Patients with SPD were characterized by empathic dysfunction with regard to others’ negative feelings, which was associated with lower indices of social support (Ripoll et al., 2013). It is important to note the inconsistency of social cognition impairment in schizophrenia spectrum disorders. Respondents with high schizotypal traits displayed a contradiction between low cognitive empathy (ability to shift from the first-person perspective to third-person perspective and back) and high affective empathy (perceptual accuracy for negative cues) (Kallai et al., 2019; Lindeman, Svedholm-Häkkinen, & Lipsanen, 2015). Studies of the Theory of Mind (ToM) in patients with SPD also demonstrate inconsistent findings. While some studies showed ToM impairment in unaffected relatives of schizophrenia patients (Bora & Pantelis, 2013), studies of individuals with psychometrically defined schizotypy (Morrison, Brown, & Cohen, 2013), studies of individuals with elevated positive schizotypy scores (Pflum, Gooding, & White, 2013), and other studies found no significant differences between respondents with schizotypal–schizoid personality disorders and controls in any of the ToM measures (Booules-Katri, Pedreño, Navarro, Pamias, & Obiols, 2019).

The cultural-historical approach discusses the systemic structure of mental activity in schizotypal disorders, and the significant role of culture in accepting cognitive-perceptual symptoms of SPD as normative experiences (Fonseca-Pedrero et al., 2018). This approach also discusses the relations of personal components (low ethnic identity, low self-concept clarity) and cognitive components (aberrant salience) of mental activity (Cicero & Cohn, 2018). A specific relationship was established in patients between affective components (“emotional investment in relationships”, “affective tone of relationships”, empathy) and cognitive components (“Complexity of representations”, “understanding of social causality”) of mentalization (Sokolova & Andreyuk, 2018; Sokolova, Andreyuk, & Ryzhov, 2018), which emphasizes the importance of interpersonal context for understanding impairment of cognition in SPD. Various studies point out the unique role of stressful events in social relations (college enrollment, adaptation to a new social environment, external requirements, etc.) for the manifestation of symptoms of SPD (Geng et al., 2013; Quide et al., 2018).

Therefore, SPD is characterized by destabilization of mental activity in situations of social interaction (Herpertz & Bertsch, 2014), which is a common pattern in personality disorders. Patients with different types of personality disorders display in social situations disturbances of emotional regulation and control, and of executive functions; less ambiguity tolerance, loss of internal consistency and sense of self-coherence (Gawda, Bernacka & Gawda, 2016; Giakoumaki, 2012; Osma, García-Palacios, Botella, & Barrad, 2014).

Thus, the investigation of visual perceptual functions in SPD requires the development of a particular methodological approach that explores the processes of mental activity in an expanded form. This approach is based on the close relationship between cognitive performance, and emotional and personality features. It includes methods that allow the researcher to vary the emotional intensity of stimuli, increase the degree of uncertainty, change instructions for tasks, and use different manners of stimulus presentation. Such an organization of an experiment is more sensitive and informative with regard to the structure of mental activity and the specific characteristics of executive functions of patients with SPD. Based on the principle of cognitive-affective unity, this approach proved its effectiveness in the investigation of hysterical personality disorder (Tkhostov & Vinogradova, 2013).

The study of interdependence, different aspects of cognitive performance, emotional and personality features, represents an integrative approach to the interpretation of experimental data. This methodology allows us to overcome limitations of the description of cognitive impairment in personality disorder at the level of separate phenomena (Duff & Kinderman, 2006) and provides a complete approach at the level of factor constellations (primary and secondary disturbances, compensatory mechanisms) (Chepeliuk & Vinogradova, 2018).

The crucial role of the cognitive-perceptual and disorganization criteria of SPD identified in clinical studies, as well as the significance of special conditions of manifestation of cognitive disturbances (an increase of emotional stress, visual perceptual characteristics of stimuli) indicate the prospects for psychological study of strategies for solving visual perceptual tasks for better understanding of the psychological mechanisms of cognitive impairment in SPD. The inconsistency of visual perceptual productivity, and the ability to find solutions corresponding to the norm differentiate SPD from schizophrenia. These peculiarities of visual perceptual processes in SPD align with the cognitive functions in personality disorders. At the same time, it is important to create conditions in which discrepancies among different types of severe personality disorders (for instance, in compensatory strategies, evaluative abilities, manner of performance) could be revealed. In our experimental study, we elaborated the conditions of different degrees of uncertainty, social regulation of answers, and complexity of stimuli to detect which is more sensitive in SPD patients. We have proposed that inconsistency of cognitive functioning productivity in SPD is manifested in steady alteration of conditions and the necessity to find new ways to adapt. These manifestations are differentiated from those in other severe personality disorders (such as obsessive-compulsive personality disorder).

This study aimed to research the performance of visual perceptual tasks in patients with SPD, their ability to create and use compensatory strategies, conditions of normative performance and manifestation of impairments, and their severity.

## Methods

### Participants

The clinical groups comprised 39 patients with SPD (F21.8 in ICD-10) aged 18–55 (experimental group) and 36 patients with obsessive-compulsive personality disorder aged 18–56 (F60.5 in ICD-10, OCPD). The clinical comparison group (OCPD) is more severe than other types of personality disorders. This allows for comparability of results of cognitive tests in patients with SPD and OCPD, to elucidate similarities and differences in psychological mechanisms of performance of visual perceptual tasks in SPD and other personality disorders.

The control group consisted of 102 healthy individuals between the ages of 18 and 54. Patients were recruited from mental health clinics in Moscow (Kannabikh State Psychiatric Hospital and International Institute of Psychosomatic Health). They were consulted by psychiatrists and signed an informed consent agreement. Patients with personality disorders and normal controls had an equivalent educational level, except in the group of patients with OCPD, who generally had higher education (*p* = 0.02) (*Table 1*). All patients were examined before the treatment.

**Table 1:** Socio-demographic characteristics of respondents

Characteristics		Subjects		
		Patients with SPD	Patients with OCPD	Healthy individuals
Age (Mean ± Standard Deviation)		29.23 ± 8.39 years	31.89 ± 9. 70 years	28.18 ± 8.48 years
Gender	Female	22 (56%)	26 (68%)	54 (53%)
	Male	17 (44%)	12 (32%)	48 (47%)
Education	Higher education	22 (56%)	30 (83%)	58 (55%)
	Incomplete higher education	12 (31%)	5 (14%)	29 (29%)
	Secondary education	5 (13%)	1 (3%)	18 (16%)

### Procedure

There were two series of visual perceptual problem-solving tests (visual perceptual test). The embedded figures of Witkin and Goldstein (Witkin, 1950) were used as stimuli. In Series I, complex figures were covered by eight simple figures and the subject had to decide whether the complex figure contains the simple one (for all 96 trials), without feedback from the experimenter. In Series II, each trial showed two complex figures simultaneously, to increase the visual perceptual load (for all 96 trials).

Three types of instructions were used. The first one was vague ( “Do you think this figure is in a complex one?” ) and was presented after the first simple figure had been shown alongside the first complex figure in Series I. The second instruction provided verbal clues about the criteria for correct answers: “Every simple figure is not necessarily embedded within every complex one, but if you find a simple figure, it should be the same as the one in the model”. This verbal clue was presented before a demonstration of the second complex figure in Series I. The third instruction involved visual perceptual cues in Series II, when one of the two simultaneously presented complex figures could be considered as a corrector for the answers. Thus, the procedure provides the opportunity to moderate uncertainty of stimuli and experimental conditions.

The analysis of performance of visual perceptual tasks included the number of correct answers in the different types of instructions.

To analyze different aspects of the cognitive functions of patients with SPD, the study included the WAIS-R test (Wechsler Adult Intelligence Scale, revised form), with six verbal and five performance subtests (Filimonenko & Timofeev, 2006).

Statistical significance was ascertained by Student’s t-test for comparing independent groups, the Spearman rank correlation coefficient, a two-way 3×3 factorial analysis of variance, and Fisher’s exact test to compare categorical variables.

## Results

### Performance of Series I and Series II

Visual Perceptual Tasks in Patients with Schizotypal Personality Disorder, Healthy Subjects, and Patients with Obsessive-Compulsive Personality Disorder.

Table 2 shows that the significance values of group and type of instruction are equal, p = .096, so there is no interaction effect by group and type of instruction of visual perceptual tasks on the number of right answers in Series I and II. ANOVA showed a significant effect of group and a significant effect of type of instruction on the number of right answers in Series I and II.

**Table 2:** Factorial ANOVA calculation results (Dependent variable: right answers in Series I and II)

Source of variance	Type of Squares III Sum	Df	Square Mean	F	Signif.
Corrected model	18.938^ª^	8	2.367	8.103	.000
Intercept	84.680	1	84.680	289.845	.000
Group	6.092	2	3.046	10.426	.000
Type of instruction	8.960	2	4.480	15.334	.000
Group × type of instruction	2.321	4	.580	1.986	.096
Error	118.908	407	.292		
Total	250.000	416			
Corrected total	137.846	415			

*Note. ^ª^ R^2^ = .137 (Adjusted R^2^ = .120)*

**Figure 1.**
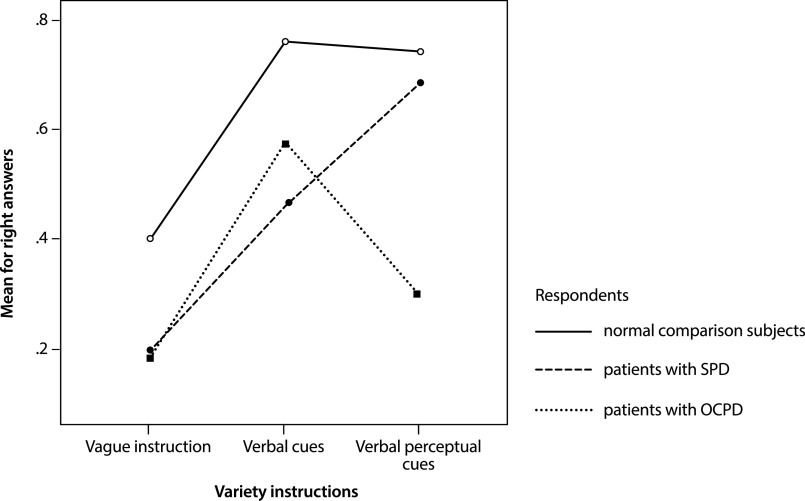
The number of right answers with different types of instruction in Series I and II for visual perceptual tasks with patients with personality disorders and healthy controls

*Figure 1* shows the values of the correct answers for the different types of instructions. The data shows that all subjects had the smallest number of answers when the key figure was presented with “vague” instructions. However, the healthy individuals had significantly more correct answers with “vague” instructions compared to the patients with personality disorders (*Table 3*). Patients with SPD had significantly fewer correct answers with verbal cues in comparison with the healthy subjects (*Table 3*). OCPD patients had intermediate outcomes with verbal cues, with more right answers than SPD patients and less than the healthy controls, without significant differences with either the SPD patients or healthy subjects (*Figure 1*). This result indicates the possibility of increasing the productivity of performance of visual perceptual tasks by patients with OCPD at the level of standard indicators, when the degree of uncertainty of the stimulus was reduced.

**Table 3:** Average of correct answers within different instructions for visual perceptual tasks within healthy subjects and patients with personality disorders in Series I and II.

Type of instruction	Respondents	Mean ± Standard Deviation	Proportion of the answers without correct figure/with correct figure
Vague instruction	Healthy subjects	0.40 ± 0.49*^3^**^2^	64% / 36%
	Patients with SPD	0.19 ± 0.09**^1^	80% / 20%
	Patients with OCPD	0.18 ± 0.39*^1^	81% / 19%
Verbal cues	Healthy subjects	0.76 ± 0.43**^2^	27% / 73%
	Patients with SPD	0.46 ± 0.51**^1^	57% / 43%
	Patients with OCPD	0.58 ± 0.50	47% / 53%
Visual perceptualcues	Healthy subjects	0.74 ± 0.72*^3^	50% / 50%
	Patients with SPD	0.69 ± 0.72*^3^	46% / 54%
	Patients with OCPD	0.30 ± 0.52*^12^	74% / 26%

Note. Fisher’s test for comparing: ^1^healthy subjects; ^2^patients with SPD; ^3^patients with OCPD. *p ≤ .05, **p ≤ .01

In Series II, with visual perceptual cues, there was an equal percentage of healthy subjects who found the key figures and of those who could not. The same was observed in patients with SPD, with slightly more patients who found the key figures in the pair of complex figures (*Table 3*). SPD patients had no significant differences in the number of correct answers in comparison with the controls (*Figure 1*). Patients with OCPD had significantly fewer correct answers with visual perceptual cues in comparison with healthy individuals (*Table 3*).

### Comparison of Results of the Wechsler Adult Intelligence Scale (WAIS-R) in Patients with SPD and Healthy Subjects

To further illuminate the performance of visual perceptual tasks, nonverbal WAIS-R subtests were analyzed for patients with SPD in comparison with healthy subjects. *Table 4* shows that the scores on the WAIS-R for patients with SPD were significantly different from those of healthy individuals. The patients scored significantly lower on nonverbal subtests such as the “Digit Symbol” (*p* ≤ .05), the “Picture Completion” (*p* ≤ .01), and the “Block Design” (*p* ≤ .01). Patients with SPD managed to fill in the same number of digits on the Digit Symbol subtest as the healthy subjects in the allotted time, but they displayed differences in the quality of writing of the symbols. The patients more often had a change in the slope or character size, an incorrect alignment of lines in the symbol, or extra lines.

**Table 4:** Comparison between WAIS-R scores of patients with personality disorders and healthy subjects

WAIS-R subtests	Participants	Mean ± SD
Information	Healthy subjects	13.65 ± 3.22**^2^
	Patients with SPD	11.56 ± 3.71**^1^*^3^
	Patients with OCPD	13.27 ± 1.94*^2^
Comprehension	Healthy subjects	13.10 ± 2.96**^2^
	Patients with SPD	11.16 ± 2.46**^1^*^3^
	Patients with OCPD	12.72 ± 2.43*^2^
Arithmetic	Healthy subjects	11.02 ± 2.67*^2^
	Patients with SPD	9.97 ± 2.65*^1^
	Patients with OCPD	10.48 ± 3.22
Similarities	Healthy subjects	13.53 ± 2.18
	Patients with SPD	13.03 ± 2.23
	Patients with OCPD	13.57 ± 2.25
Digit Span	Healthy subjects	11.69 ± 2.45*^2^**^3^
	Patients with SPD	10.61 ± 2.45*^1^
	Patients with OCPD	10.00 ± 2.73**^1^
Vocabulary	Healthy subjects	14.22 ± 2.52*^2^
	Patients with SPD	13.26 ± 2.21*^1^
	Patients with OCPD	13.94 ± 2.30
Sum of verbal scores	Healthy subjects	77.18 ± 10.88**^2^
	Patients with SPD	770.64 ± 11.55**^1^
	Patients with OCPD	73.85 ± 8.47
Digit Symbol	Healthy subjects	10.55 ± 2.73*^23^
	Patients with SPD	9.27 ± 2.52*^1^
	Patients with OCPD	9.48 ± 1.95*^1^
Picture Completion	Healthy subjects	12.02 ± 1.92**^23^
	Patients with SPD	110.94 ± 2.10**^1^
	Patients with OCPD	10.34 ± 1.94**^1^
Block Design	Healthy subjects	13.83 ± 2.56**^23^
	Patients with SPD	12.06 ± 3.29**^1^
	Patients with OCPD	12.03 ± 2.57**^1^
Picture Arrangement	Healthy subjects	10.24 ± 2.00
	Patients with SPD	9.53 ± 2.67
	Patients with OCPD	9.78 ± 1.43
Object Assembly	Healthy subjects	7.68 ± 2.13
	Patients with SPD	8.11 ± 2.61
	Patients with OCPD	8.22 ± 2.52
Sum of performance scores	Healthy subjects	54.31 ± 6.63*^2^**^3^
	Patients with SPD	49.86 ± 10.02*^1^
	Patients with OCPD	50.13 ± 6.58**^1^
Full Scale Scores	Healthy subjects	131.60 ± 15.32**^2^*^3^
	Patients with SPD	120.94 ± 19.88**^1^
	Patients with OCPD	123.81 ± 11.86*^1^

Note. Student’s t-test for comparing independent groups: ^1^healthy subjects; ^2^patients with SPD; ^3^patients with OCPD; *p ≤ .05, **p ≤ .01.

The lower scores in the Block Design subtest in patients with SPD was associated with difficulty analyzing parts within the whole, when this was required to compare the results of their performance with an externally provided sample.

Patients with SPD had lower productivity in the Picture Completion subtest because of a tendency to focus on insignificant, peculiar aspects of the stimuli.

An important characteristic of the cognitive processes of patients with SPD was the significantly lower score on WAIS-R verbal subtests. Analysis of performance of verbal tasks by patients with SPD allowed us to elucidate the general psychological mechanisms, which can also determine the impairment of visual perceptual processes in SPD.

Patients with SPD differed statistically from healthy subjects in the performance of the Information (*p* ≤ .01), Comprehension (*p* ≤ .01), Arithmetic (*p* ≤ .05), Digit Span (*p* ≤ .05), and Vocabulary (*p* ≤ .05) verbal subtests.

On the Information subtest, SPD patients showed a paradoxical combination of difficulty in extracting information reinforced by experience, and ease answering the most difficult questions.

The lower scores in the Comprehension subtest in patients with SPD was caused by disregarding the social context, relying solely on the subjective manner of behavior in stimulus situations (egocentric position). In some cases, the answers of patients with SPD included both egocentric solutions and standard social criteria. When conveying the figurative meaning of non-frequent expressions, SPD patients tended to resort to magical meanings combined with situation-based generalizations. It should be noted that the patients could give the correct answers in tasks where they were asked to explain the figurative meaning of common proverbs and expressions, which suggests the ability of these patients to operate with conventionalized abstract meaning.

Patients with SPD had lower scores on the Arithmetic subtest in comparison with healthy subjects, which can be explained by the former’s inconsistent productivity in counting operations. These patients, when faced with difficulties in tasks with one-phase counting, did not attempt to find an answer. More rarely, patients with SPD expressed unwillingness to count. By contrast, the patients performed well on easy tasks on the Arithmetic subtest with two-phase counting, and obtained additional points for speed in these tasks. In the most complicated tasks of the Arithmetic subtest, patients with SPD easily substituted a correct description of the necessary actions, thereby avoiding counting operations.

Comparison of Performance on the Wechsler Adult Intelligence Scale (WAIS-R) in Patients with OCPD and Healthy Subjects

*Table 4* shows a significant difference between the WAIS-R performance by patients with OCPD and healthy individuals only in the Digit Span verbal subtest (*p* ≤ .01). However, in nonverbal subtests, the results of patients with OCPD were significantly lower than with healthy subjects (in the Digit Symbol (*p* ≤ .05), in the Picture Completion (*p* ≤ .01) and in the Block Design (*p* ≤ .01) subtests).

The lower scores on the Digit Span subtest were associated with a shift of patients’ attention from the instruction for the task to thoughts of failure, greater doubts about one’s own performance, abilities, etc. Patients with OCPD demonstrated on the Digit Symbol subtest the intention to accurately imitate the symbols specified by the instruction, which led to an excessive focus of attention on verifying the result. They also corrected their mistakes, which slowed their performance. Preoccupation with details made the patients with OCPD take a long time searching for answers in the Picture Completion subtest, going beyond the time allocated to each item.

Patients with OCPD gave a detailed analysis of the parts that presented difficulties when synthesizing them into a whole, were unable to revise their work method, doubted the correctness of the process, and often canceled the correct answers, which reduced their productivity in the Block Design nonverbal subtest.

Patients with OCPD had significantly higher scores than patients with SPD on the Information (*p* ≤ .05) and Comprehension (*p* ≤ .05) verbal subtests. Analysis of the performance of nonverbal subtests by patients with different types of personality disorders revealed no significant differences.

Correlations of Parameters of the Wechsler Adult Intelligence Scale and of the Visual Perceptual Tasks in Patients with Different Types of Personality Disorders

In healthy subjects and patients with OCPD, there were no significant correlations between the number of right answers on the Series I and Series II visual perceptual tasks with different types of instructions, and their scores on WAIS subtests.

In patients with SPD there were significant positive correlations between the number of correct answers under the condition of visual perceptual cues, and the Sum of Performance scores (*r* = 0.37, *p* ≤ .05) and scores on the Object Assembly subtest (*r* = 0.49, *p* ≤ .01).

## Discussion

The study revealed that patients with different types of personality disorders had similarities in their profiles of reduced effectiveness in nonverbal tasks in comparison with standard indicators. The lack of differences in performance of nonverbal tasks between patients with schizotypal and obsessive-compulsive personality disorders suggests that working with visual perceptual information is the most sensitive to deviations from standard indicators in patients with personality disorders.

Performance of the Series I and Series II visual perceptual tasks with vague instructions, and with verbal and visual perceptual cues, allows us to elucidate the psychological mechanisms behind differences in cognitive impairment and compensatory strategies in personality disorders. Although in conditions of high uncertainty with vague instruction, patients with SPD and OCPD did not differ from healthy controls, the introduction of verbal cues in Series I brought to the fore the principal impairments in SPD patients (lack of social regulation, inability to change the manner of decision-making by comparing their solutions with the model), and the adaptive ability of patients with OCPD. The latter were able to develop their own effective criteria based on the verbal cue (using the verbal cue “to be exactly like the model”) in searching for the key figures embedded in the complex ones.

In the performance of Series II tasks with visual perceptual cues, the situation is the opposite. SPD patients could switch their attention to the second figure and treat it as a corrector to their own solutions, so, they had standard results, whereas patients with OCPD demonstrated a significant decrease in performance of visual perceptual tasks when two complex figures are presented simultaneously. These findings indicate the abilities of patients with SPD to find a new way of adapting when the conditions of performance were changed and to follow it effectively. This corresponds to the fact that patients with SPD had no discrepancies with normative indicators when working on nonverbal tasks with visually presented social context (the Picture Arrangement subtest) and concrete content (the Object Assembly subtest). This fact suggests that patients with SPD had no decrease in social regulation of activity in case of direct visual support of the social context. At the same time, patients with SPD display lower productivity in verbal tasks associated with a social context. Therefore, among nonverbal tasks, the performance of tasks with abstract visual perceptual information is the most sensitive to impairments in patients with SPD. This result is consistent with the significant role of cognitive-perceptual criteria for diagnosis of SPD identified in clinical studies (Zouraraki et al., 2017).

The decline in most cognitive parameters of the Wechsler Adult Intelligence Scale in patients with SPD corresponds to data from meta-analyses of cognitive impairment in schizophrenia spectrum disorder (Carruthers et al., 2019) and suggests greater severity of cognitive impairment in SPD patients in comparison with patients with OCPD.

SPD patients’ inconsistent productivity in performing verbal tasks was caused by difficulties in inhibiting subjectively significant responses when socio-deterministic formal criteria were available for making judgments; the stability of a subjective manner of performance and inability to revise it; vagueness in definition of concepts; and slipping into subjective associations with the stimuli. OCPD patients are characterized by relatively intact ability to solve verbal tasks, as evidenced by lower scores than normative on only one of the verbal subtests.

Although patients with SPD and OCPD did not differ significantly in their scores on nonverbal tasks, the qualitative analysis revealed the principal differences in the psychological mechanisms of their impairments. In SPD patients, the lower scores on nonverbal subtests were a result of low orientation toward the model, ease of subjective transformation of the targets, and impairment of the selectivity of cognitive processes. The latter is specific for patients with schizophrenic spectrum disorders. This impairment is characterized by a tendency to rely on insignificant, bizarre, unusual attributes of concepts (Kritskaya & Meleshko, 2015; Kritskaya, Meleshko, & Polyakov, 1991). The lower productivity on nonverbal subtests of patients with OCPD was associated with their detailed analysis of parts that present difficulties in their synthesis, preoccupation with details, inability to revise their work method, and doubts about their accuracy, often with the rejection of correct answers.

For healthy subjects and patients with OCPD, there was no association in Series I and Series II between productivity in performing visual perceptual tasks under conditions of different instructions, and the parameters on the intelligence scale, whereas in SPD patients, the productivity of the search for figures under condition of visual perceptual cues was associated with nonverbal subtests and the integrative performance score.

## Conclusion

The patients with SPD had more severe cognitive impairment than the OCPD patients. The former had lower productivity in performance of verbal and nonverbal tasks. Patients with OCPD had difficulties only in the performance of nonverbal tasks, whereas in verbal tasks they had scores corresponding to the normative.

It is important to note the specific mechanisms of impaired cognitive functioning in SPD. The basic impairment in these patients was associated with difficulties in inhibition of bizarre responses, stability of the subjective manner of performance and inability to revise it, low orientation to the model, and slipping into subjective associations with the stimuli. In OCPD it was associated with a shift of patients’ attention from the instruction about the task to thoughts of failure, strengthening of doubts, and excessive focus on checking one’s performance.

Varying the degree of uncertainty of the visual perceptual tasks with abstract stimuli made it possible to reveal the specific compensatory strategies of patients with SPD, which help to increase the productivity of the performance. So, under the condition of visual perceptual cues, these patients could achieve normative results, whereas patients with OCPD demonstrated lower productivity. These facts also suggest that the performance of visual perceptual tasks in patients with SPD is characterized by inconsistent productivity and the availability of compensatory strategies that increase the performance to normative levels.

This experimental study has some clinical implications. First, these findings broaden the pathopsychological picture of impairments of visual perceptual processes and compensatory strategies of patients with SPD. Second, it should be useful for differential diagnostics of severe personality disorders, SPD, and other schizophrenia-spectrum disorders. Furthermore, these results contribute to addressing complex issues of social and daily-life adaptation, occupational activity, and productivity of SPD patients.

## Limitations

The current study had several limitations that should be addressed in future research. First, it might be informative to compare the results of cognitive tasks of patients with SPD and schizophrenic patients (pseudoneurotic type), to discuss specific and general impairment of cognitive productivity in schizophrenia-spectrum disorders. Second, it would be promising to discuss the duration of clinical symptoms and their association with the change or intactness of cognitive productivity in the performance of the visual perceptual tasks in patients with SPD.
